# Emotion Analysis of Arabic Tweets: Language Models and Available Resources

**DOI:** 10.3389/frai.2022.843038

**Published:** 2022-03-30

**Authors:** Ghadah Alqahtani, Abdulrahman Alothaim

**Affiliations:** Information Systems Department, College of Computer and Information Sciences, King Saud University, Riyadh, Saudi Arabia

**Keywords:** emotion analysis, language models, natural language processing, Arabic tweets, social emotion

## Abstract

One of the most popular social media platforms is Twitter. Emotion analysis and classification of tweets have become a significant research topic recently. The Arabic language faces challenges for emotion classification on Twitter, requiring more preprocessing than other languages. This article provides a practical overview and detailed description of a material that can help in developing an Arabic language model for emotion classification of Arabic tweets. An emotion classification of Arabic tweets using NLP, overall current practical practices, and available resources are highlighted to provide a guideline and overview sight to facilitate future studies. Finally, the article presents some challenges and issues that can be future research directions.

## Introduction

The amount of subjective information available on the internet has increased tremendously in recent years. Especially after the coronavirus disease-2019 (COVID-19) pandemic, the number of users who generated and shared content in various online platforms increased rapidly. Furthermore, social media platforms are a useful source of information, since they give users the ability to discuss and share their opinions and emotions. There was no way to know what people thought about certain items, services, or even events until these platforms arose. This valuable information is accessible today for anyone because of social media platforms such as Twitter, where individuals regularly share their ideas openly and even use other people's opinions to influence their actions.

Opinions of users can be analyzed to give feedback on specific services, events, or products. Analysis of users' opinions can be performed with the use of natural language processing (NLP), text mining, computational linguistics, and machine learning algorithms for detecting, analyzing, and classifying human's feelings, opinions, sentiments, evaluations, appraisals, attitudes, and emotions (Shukla and Shukla, 2015). Sentiment analysis (SA) can be described as an NLP task aiming to identify a subjective content that contains feeling and sentiment, which are classified as positive, negative, or neutral (Singh et al., 2013). Furthermore, textual data can be divided into two categories: facts and opinions. Facts are objective statements about things, events, and their characteristics. Opinions are usually subjective expressions of people's sentiments, assessments, or feelings concerning entities, events, and their attributes. When building a model for sentiment analysis and classification, some of the literature considering focusing on the polarity of opinion (positive or negative). On the other hand, a number of research studies consider objective expression as a neutral class in SA to build a classifier able to distinguish between subjective and objective opinions. Correspondingly, the field of emotion analysis and classification differs from that of SA. It goes deeper than SA in describing a person's emotions. Emotion analysis allows for more detailed emotions to be analyzed and classified (Al-A'abed and Al-Ayyoub, 2016). Emotion is a part of the nervous system that is linked to an emotional state like joy, anger, or sadness. The process of emotion analysis is identifying whether the content of a text contains emotions, and detecting that emotion by classifying it into a suitable emotion category.

Published information on Twitter comes in the shape of tweets that contain text, in various languages, and users post more than 400 million tweets every day (Wikarsa and Thahir, 2015). Posted tweets contain users' emotions and feelings in different languages. Analysis of emotions in tweets has many difficulties, because these tweets have many grammatical errors, misspellings, slang, social shortcuts, and multimedia contents. Many researchers study emotions in English language tweets, but none of them classify the emotions in Arabic language tweets because of its challenges. Most feeling analyses of Arabic tweets are focused on classifying a sentiment into a positive or a negative feeling.

The Arabic language, until today, lacks in studies and resources in the field of social emotion analysis, and models for extracting and classifying emotions in Arabic tweets will be useful in a variety of fields, including improving E-learning applications, assisting psychologists in detection of terrorist behavior, improving customer service, improving product quality, and many others.

Language is complex, and processing it is computationally challenging. Words are essential building blocks of language. In natural language processing (NLP), words must be converted to a numerical format to aid machines in understanding the language by giving an appropriate representation. As a result, language models evolved, and vector space models were used, in which words are represented by numbers in the form of vectors. In recent years, the emergence of contextualized language representations has ushered in a revolution in NLP. The evolution of NLP language models was from simple frequency counts like bag of words, n-grams, and term frequency-inverse document frequency (TF–IDF) to more advanced representations like Word2vec (Mikolov et al., 2013), GloVe (Pennington et al., 2014), and FastText (Bojanowski et al., 2017), which use neural networks to learn features unsupervised in big data sets. Despite the fact that these early models have made significant progress, there is still lack of contextualized information.

Recently, with the rise of the neural attention-based deep architecture, referred to as transformer architecture, the discipline of NLP has just seen major progress (Vaswani et al., 2017). Transformers excel when dealing with long-term text dependencies. Several advanced architectures have been proposed since the release of initial transformer models: BERT (Devlin et al., 2018), XLNet (Yang et al., 2019), GPT (Radford et al., 2018), RoBERTa (Liu et al., 2019), and ALBERT (Lan et al., 2019). Typically, transformers and their variants are pretrained on large unlabeled language data sets and corpora, such as the web corpus and Wikipedia, to learn word representations that are contextual, which means each word has different representations depending on the context it appears in, allowing for the capture of words used in a variety of contexts. Furthermore, pre-trained language models (PLMs) can be fine-tuned and used on a downstream task. In practically every NLP task, fine-tuning a PLM in a variety of downstream tasks has resulted in significant improvement in performance (Qiu et al., 2020).

The evolution of language modeling for emotion classification tasks is our focus in this study to give researchers in these field an overview, which can be a starting point or a guide, since we will discuss available resources and current practices specially for the Arabic language. The contributions of this article are as follows:

- We compare the model's used transformer in an emotion classification task for Arabic language.
- We went through the current status of the Arabic NLP data sets for emotion classification, and discovered the methodology and practices to build an emotion tweet data set.
- We provide a detail of available tools, resources, and open source python libraries that can be used for building a model to classify emotions in Arabic tweets.

The article is structured as follows: Section Related Studies and Background looks into related studies in the literature; Section Emotion Analysis of Arabic Tweets goes through some of existing emotion models for Arabic tweets, highlights the Arabic PLM available for fine-tuning, and summarizes available resources and tools; Section Issues and Challenges in Emotion Analysis of Arabic Tweets discusses tissues and challenges in the emotion analysis of Arabic tweets.

## Related Studies and Background

To the best of our knowledge, previous studies that conducted reviews for available resources and current practices were either in the English language or the attention was on specific tasks such as question answering with a specific language model (Mahdi, 2021) or text classification (Cruz and Cheng, 2020) and machine translation (Harrat et al., 2019).

There has been a number of research studies in the field of Arabic language focused on the analysis of articles in the field of NLP. For instance, Mohammad (2020) reviewed the literature with over 1.1 million article information data sets acquired from Google Scholar. A comprehensive study by Farha and Magdy (2021) provided a thorough comparative analysis of available approaches on a sentiment analysis task. Furthermore, study survey the NLP literature on dialectical Arabic data sets that were available. Shoufan and Alameri (2015) evaluated the dialectical Arabic NLP literature, and the work can be used as a reference to find relevant contributions for distinct Arabic dialects that address specific NLP aspects. However, this research was focused only on data sets for Arabic dialects that were accessible.

Currently, a practical overview and detailed description of materials that can help in developing and coding an Arabic language model for emotion classification of Arabic tweets in the literature are not available. The emotion classification of Arabic tweets using NLP overall current practical practices and available resources needs to be considered to provide a guideline and overview sight to facilitate future studies. Throughout the next sections, we will give an overview of related terms and concepts starting with the language nature of the data down to the main focus of our study, Arabic language models.

### Arabic Language

The Arabic language is both difficult and fascinating. It is fascinating because of its history, strategic significance of its people and the region they inhabit, and its cultural and literary heritage. It is also difficult because of its complicated language structure. Classical Arabic has remained unmodified, intelligible, and functional for centuries at a historical level. In terms of culture, the Arabic language is linked to Islam and a prestigious body of literature. It is the native language of more than 400 million people (Boudad et al., 2018) who live in a strategic region with vast oil supplies that are vital to the global economy. Classical Arabic (CA) was the language spoken by Arabs over fourteen centuries ago, whereas Modern Standard Arabic (MSA) is an evolving variation of Arabic with constant borrowings and innovations, demonstrating how Arabic reinvents itself to fit the changing demands of its speakers (Alhawarat et al., 2015). According to that, the importance of the Arabic language cannot be overstated. Not only is it used by hundreds of millions of people, its online presence in terms of users and content is rapidly growing. Furthermore, Arabic has a variety of different characteristics that make automated handling and comprehension of Arabic text complex and fascinating. The Arabic language is characterized as being morphologically rich and complex. Multiple prefixes, suffixes, and infixes can be added to a single stem in Arabic words.

In addition, there are two different types of morphology, derivational and inflectional. The derivational morphology is prevalent, allowing numerous words to be derived from a single stem. Inflectional morphology, on the other hand, can combine numerous morphemes into a single stem to produce considerably longer and more complicated words. As a result, there are about 5,000 or more different forms of verbs. This diverse morphology gave rise to a wide range of dialects throughout the Arabic world. Furthermore, the language's complexity makes language modeling difficult in general. As we have mentioned above, there are three major types of Arabic: CA, a form of Arabic language used in the Quran and is formal and textual (Sharaf and Atwell, 2012); MSA, which is used for writing and formal conversations; last but not least, Arabic dialect (AD), which is used in our daily lives and informal textual conversations. More details about the Arabic language used in social media are given in the following sections.

#### Arabic Language in Social Media

More than 400 million people speak Arabic, which is the official language of 22 nations. It is the fourth most widely used language on the Internet (Boudad et al., 2018). Languages used in social media, for example Twitter, differ significantly from that utilized in other platforms such as Wikipedia. Nonlinguistic contents are written out, such as laughter, sound representations, and emoticons, and the vocabulary is informal with intentional deviations from standard orthography such as repeated letters for emphasis errors, and nonstandard abbreviations are widespread. This issue is aggravated in the case of Arabic social media for a variety of reasons, including the fact that Arabic dialects often used in social media are phonologically, morphologically, and lexically distinct from MSA, and they lack a standard (Habash, 2010).

Consequently, social media Arabic text has special characteristics compared to those of CA, MSA, and AD. Written text in these platforms could be a combination of all of these types, as well as non-Arabic words, samples, notations, and orthographic elements like spelling mistakes, repeated letters, or emoticons. As a result, in addition to dealing with the Arabic language's complexity concerns, other issues such as preparing the text and removing noise, and dealing with dialects and languages, need to be considered (Hegazi et al., 2021).

### Language Representation Models

Many approaches and algorithms have been developed over the years to infer vectors from text, whether at the character, word, sentence, or document level. All of the methods are intended to gain better measuring of the information's richness and make it more machine-learnable. Early language representations, such as TF–IDF, a bag of words, and n-grams, depended primarily on capturing the frequencies of word occurrences in a text. These models, however, failed to capture the words' syntactic and semantic meaning, and, thus, suffered from the constraint of high dimensionality. In addition, these language representation models were insufficient for text categorization on their own and must always be augmented with specific features created by hand, which can be a time-consuming and labor-intensive process. The shortcomings of these models led researchers to learn the representation of distributed words in a low dimension space (Bolukbasi et al., 2016).

Because of the limits of traditional feature extraction methods, other models have been proposed in the past that automatically discover representations for downstream tasks such as classification. Feature learning or representation learning refers to methods that can extract features without need for manual extraction. It is essential because the representations of an input have a significant impact on machine learning (ML) models' performance (Bengio et al., 2013). Because of prior knowledge of various ML models, word embeddings have become particularly efficient representation methods in the field of NLP to increase the performance of many downstream tasks. Because of their high capacity of representation learning, traditional feature learning approaches have been supplanted by methods based on neural networks. Word embedding is a feature learning method in which a vocabulary or a word is mapped to an N-dimensional vector. Word2Vec (Mikolov et al., 2013), Glove (Pennington et al., 2014), and FastText (Bojanowski et al., 2017) are some of the word embedding methods that have been presented. Because they contain fewer parameters than sparse vectors of explicit counts, these neural language models are easier to include as features in ML systems, and they generalize better and help minimize overfitting. Using a shallow neural network, they learn automatically from text. As a result, current NLP research has concentrated on representation-learning approaches to learn features automatically and unsupervised from inputs in order to eliminate the considerable human labor of feature creation.

Recently, NLP research has concentrated on representation learning, which is the process of autonomously learning features from inputs in an unsupervised approach. Despite the fact that these representations have improved performance on text classification tasks, they still have a number of shortcomings. For instance, they are static, shallow, and unaffected by context; each word has only one vector representation regardless of its meaning. Moreover, the out-of-vocabulary problem, which means that some terms that are not in the data set, are not represented yet.

The field of NLP has grown significantly since the introduction of transformers (Vaswani et al., 2017). Transformers are composed of various layers of encoders and decoders that contain, among other things, multiple attention head components. Transformers address the constraint of previous recurrent neural network (RNN) limitation of only looking at the previous words of a sequence by looking at all the words around the target word and assigning more weight to keywords, which is known as the self-attention mechanism. As a result, each word is represented in relation to its context, and words might have many vector representations depending on their meaning. This is in contrast to prior word embedding methods, which used a single vector representation for each word regardless of context or meaning. Transformers also have the ability to analyze text in parallel rather than sequentially, which speeds up the process. The current emergence of pre-trained language models can be attributed to the combination of transformers and the concept of transfer learning. The aim is to use the knowledge of a pre-trained model while completing a different task. Many machine learning models, including deep learning models such as artificial neural networks and reinforcement learning models, can benefit from transfer learning.

The transformer's evolution and the development of various models to learn universal language representations lead to better performance and speed up convergence on downstream tasks. Following the pre-training step, the fine-tuning process refers to the adjustment of PLMs for downstream NLP tasks. A classification layer is added to this model, which uses SoftMax to compute label probabilities. These massive PLM, which have billions of parameters and have been learned from nearly all text published on the internet, have improved the state-of-the-art on nearly every downstream NLP task, such as question answering, conversational agents, and sentiment analysis (Qiu et al., 2020).

For language modeling, a transformer has been shown to be more efficient and faster than long short-term memory (LSTM) or convolutional neural network (CNN), and its basic architecture consists of encoder and decoder blocks (Vaswani et al., 2017). The size of a model is determined by whether the encoder or decoder is used alone or both by stacking multiple encoder and decoder blocks. The bidirectional encoder representations from transformers (BERT) model, for example, relies solely on the encoder and comes in two sizes: the base, which stacks 12 encoders, and the large, which stacks 24 encoders. The BERT model has pre-trained with two objectives: masked language model (MLM), when given a sentence, the model masks 15% of the words from the input randomly, then runs the entire masked sentence through the model, which must predict the masked words, and next sentence prediction (NSP), which requires the model to predict the next sentence from a given sentence. More language understanding models have been developed after BERT's introduction, including RoBERTa (Liu et al., 2019), XLNet (Yang et al., 2019), ALBERT (Lan et al., 2019), and ELECTRA (Clark et al., 2020), which have improved performance by experimenting with new pre-training approaches, updated model architectures, and larger training corpora.

## Emotion Analysis of Arabic Tweets

First, we will go through some of the existing emotion models for Arabic tweets. Following that, we will highlight the Arabic PLM available for fine-tuning. Then, we will present the corpus, lexicons, and data sets that can be used to pre-train and fine-tune the PLM for Arabic language and emotion classification as a downstream task. Finally, we will discuss other available resources and tools, which exist in the literature and may help in pre-training language models for the Arabic language and emotion classification of tweets.

### Emotion Models

Emotion detection and classification of Arabic text have recently gotten a lot of attention; however, there is a lot of work that needs to be done in this field to develop more accurate emotion detection models in social media.

In the domain of Arabic emotion analysis, previous studies have relied on lexicon-based methods (Abd Al-Aziz et al., 2015; Al-A'abed and Al-Ayyoub, 2016), ML methods (Hussien et al., 2016; Al-Khatib and El-Beltagy, 2017), and a hybrid approach combined with ML methods with textual features of contents to classify textual data. For instance, Abdullah et al. (2018) combined the lexicon-based method with a multi-criteria decision-making approach to classify text into emotion classes.

Machine learning (ML) methods can be categorized into supervised and unsupervised approaches, which are used to detect emotional expressions in text, such as happiness, sadness, and anger, automatically. Studies in the body of literature have shown that a supervised machine learning approach can outperform lexicon-based approaches (Syed, 2015). Moreover, Rabie and Sturm (2014) provided a method for detecting and classifying emotions in Arabic tweets written in both the standard and slang Egyptian dialects. Techniques for preprocessing were discussed, and the average accuracy score for all emotions was 64.3%. The authors of this article went into various points about preprocessing, although the data set utilized in this study was specific to the Egyptian revolution of 2011. Furthermore, the data set is small, with 1,605 tweets. On the other hand, the study results should be improved.

Traditional ML algorithms are constrained by the requirement that both the training and testing data sets should belong to the same feature space and justify the same distribution (Weiss et al., 2016). Transfer learning, on the other hand, allows for a wide range of domains, distributions, and tasks utilized to train and test models (Lu et al., 2015). The primary objective of transfer learning (TL) is to develop a high-performing model in a target domain using data from a source domain. The model's performance is improved by incorporating knowledge from a related area (Weiss et al., 2016). When obtaining training data sets is expensive, difficult, or impossible, the technique might be used. Training and test data for TL must not be independent and identically, to allow for evaluation of TL in case there is lack of training data (Tan et al., 2018). TL has been investigated for classification, clustering, and regression problems in traditional ML. Because DL is such a powerful ML technique, it is crucial to look into deep TL applications (Tan et al., 2018).

After analyzing the literature on Arabic emotion classification of tweets, we discovered that the majority of previous studies utilized either traditional ML with features or emotion lexicons, or deep learning algorithms such as Word2vec or FastText, which use non-contextual embeddings. Only two studies, however, attempted to use PLMs and TL for the task of Arabic emotion classification. The first model was a BERT-based model named MARBERT (Abdul-Mageed et al., 2020), which was trained on tweets, and social meaning tasks were considered in the fine-tuning. The social meaning tasks included emotion detection. The second model was the QARiB model (Abdelali et al., 2021) that was trained on a collection of over 420 million tweets, 180 million sentences of text that was a combination from Arabic GigaWord, Abulkhair Arabic Corpus, and OPUS. The emotion detection task was performed on the QARiB and four BERT-based models.

Until now, emotion analysis of Arabic tweets still requires more effort, especially considering the benefits of pre-training and fine-tuning a model for these tasks can provide. A significant number of Arabic PLMs were found in the literature, and we will go through them in detail in the next section.

### Pre-Trained Language Models for Arabic

Construction of numerous bidirectional transformer types, particularly for Arabic, has seen a major revolution in the last few years. They serve as effective transfer learning techniques for a variety of NLP tasks such as text classification, named entity recognition (NER), and question answering (QA). While fine-tuning transformer-based models, researchers produced cutting-edge outcomes on a variety of downstream NLP tasks.

In terms of contextual language models for non-English languages, a multilingual BERT model was developed that was trained on Wikipedia dumps of over 100 languages such as Arabic. The literature, on the other hand, demonstrates that pre-training monolingual BERT performs better than multilingual BERT. The AraBERT (Antoun et al., 2020a) model, which is a BERT-based model pre-trained for Arabic language, and a pre-trained contextualized text representation model. The model was trained on Arabic Wiki dumps, the 1.5 billion-word Arabic Corpus, the OSIAN Corpus, Assafir news articles, and four additional manually crawled news websites. Furthermore, it is available in several versions, including AraBERT_v1_, AraBERT_v02_, and AraBERT_v2_, and distinction is in the usage of pre-segmented text in AraBERT_v1_, where prefixes and suffixes were split using FarasaSegmenter (Abdelali et al., 2016), an Arabic-specific text segmenter. AraBERT's applicability to tasks involving dialects is limited, because it is pre-trained using MSA data. Three tasks were used to evaluate AraBERT: sentiment analysis, NER, and QA. Recently, AraBERT released a new version, AraBERT_v0.2Twitterbase_^1^, and large are two new models for Arabic dialects and tweets, trained by continuing the pre-training on over 60 M Arabic tweets.

Meanwhile, Safaya et al. (2020) proposed ArabicBERT, which increases the amount of corpus used in the earlier AraBERT. The models were pre-trained using the OSCAR in Arabic, a recent dump of Arabic Wikipedia, and other Arabic resources. ArabicBERT is available in four sizes depending on the size of the architecture: mini, medium, base, and large. Table 1 shows the architectures of these four versions of ArabicBERT and the earlier AraBERT. In another study on PLM for the Arabic language, a transformer architecture, ELECTRA, which contains two modules, a generator and a discriminator, was pre-trained. Usually, a discriminator is taken and fine-tuned for downstream tasks. The Arabic-specific ELECTRA provided by Antoun et al. (2020b), was trained on the same textual corpus used for AraBERT. However, we noticed that AraELECTRA is one of the top performing models while being much more efficient in its computational cost and achieves similar results to AraBERT that has doubled the number of parameters. As a result, it may be the best option when working with limited resources.

**Table 1:** Summary of Arabic pre-trained language model versions, architecture details, and training data sets.

**Arabic pre-trained language models**						
**Name**	**versions**	**Details**				**Pre-train data**
		**Layers**	**Hidden**	**Heads**	**Parameters**	
Multilingual BERT	Multilingual _Cased_	12	768	12	110M	W
	Multilingual _Uncased_	12	768	12	110M	
AraBert	AraBERT_base_	12	768	12	136M	OS, W, N
	AraBERT_large_	12	768	12	371M	
	AraBERT_Twitterbase_	12	768	12	136M	OS, W, N, T
	AraBERT_Twitterlarge_	12	768	12	371M	
ArabicBERT	ArabicBERT_Mini_	4	256	4	11M	OS, W
	ArabicBERT_Medium_	8	512	8	42M	
	ArabicBERT_Base_	12	768	12	110M	
	ArabicBERT_Large_	24	1024	16	340M	
QARiB	QARiB25_mix_	12	768	12	110M	S, N, T
AraGPT2	AraGPT2_base_	12	768	12	135M	OS, W, N
	AraGPT2_medium_	24	1024	16	370M	
	AraGPT2_large_	36	1280	20	792M	
	AraGPT2_mega_	48	1536	25	1,46B	
AraELECTRA	AraELECTRA_base_	12	256	4	60M	OS, W, N.
Arabic ALBERT	ALBERT_base_	12	768	12	12M	W, OS
	ALBERT_large_	24 layers	1024	16	18M	
	ALBERT_xlarge_	24 layers	2048	32	60M	
MARBERT	MARBERT	12 layers	256	12	160M	OS, W, N, B, T

*The pre-train data are indicated as follow: Tweets (T), Wikipedia (W), news (N), OSCAR corpus (OS), subtitles (S), and books (B)*.

Antoun et al. (2020c) presented AraGPT2, a stacked transformer decoder model trained using the causal language modeling objective, which is based on the original GPT2 (Radford et al., 2019) architecture. The model was trained using the same text corpus as AraELECTRA and AraBERT. The authors did not test GPT2 on any data sets, because it was trained using a causal language modeling objective; thus, they relied on the perplexity reported during training. As we have mentioned, AraGPT2 is trained using a causal language modeling objective, which appears to be effective for sentence completion and language generation tasks but not for classification tasks. An Arabic version of ALBERT was provided by Arabic ALBERT. This model was trained on data from the Arabic version of the OSCAR corpus and the Arabic Wikipedia. There are three variants of this model based on the number of parameters. The details of the models are shown in Table 1. The model uses parameter reduction techniques such as factorized embedding parameterization, cross-layer parameter sharing to reduce the number of parameters by 18× and faster training by 1.7×.

Arabic ALBERT^2^ provides an Arabic version of ALBERT (Lan et al., 2019). The Arabic Wikipedia and the Arabic version of the OSCAR corpus were used to train this model. This model is available in three main versions depending on the number of parameters. The model reduces the number of parameters and speeds up training using parameter reduction approaches such as factorized embedding parameterization and cross-layer parameter sharing. While we were investigating emotion analysis of Arabic tweets, we noticed that MSA is mostly utilized to train a language model for Arabic. Textual data on Twitter are frequently dialectal, with dialects lacking spelling norms and informal in nature, and may include emojis, hashtags, and user mentions. Only QARiB (Abdelali et al., 2021) and MARBERT (Abdul-Mageed et al., 2020), as mentioned above, used tweets to train transformer models and fine-tune for emotion classification tasks, and both of them are BERT-based. Moreover, even when testing on informal text, increasing the variety of training data by including both formal (MSA) and informal (AD) text is better than using informal text alone (Abdelali et al., 2021). Chowdhury et al. introduced a new Arabic BERT in (Abdelali et al., 2021) QARiB. The authors attempted to improve the model's performance by varying the training data in their study. They show that a BERT model trained on a mixture of data has considerably superior generalization capability than a BERT model trained just on MSA text in their experiments.

In a similar vein, Abdul-Mageed et al. (2020) trained MARBERT to increase its capacity to handle dialectal Arabic. They enhanced the training data with a set of 1 Billion Arabic tweets that were randomly collected. The total amount of text in the final training data set was roughly 128 GB, with almost half of it being tweets. This model has been evaluated on sentiment analysis, social meaning prediction, topic categorization, dialect identification, and named entity recognition, among other NLP tasks. MSA and AD were used to train models like MARBERT and QARiB. The performance of these models is noticeably superior to that of other similar or even larger versions, such as ArabicBERT_large_ and Arabic ALBERT_large_. However, it is worth noting that the applied prepossessing has an effect on the result of similar models. AraBERT_base_ and ArabicBERT, for example, are built on the same architecture and trained on the same data; however, AraBERT outperforms ArabicBERT. Non-Arabic words are removed during AraBERT preprocessing; however, non-Arabic words are in line with ArabicBERT data.

Furthermore, it is obvious that the training process has a significant impact on a model's performance. For classification tasks, models trained with masked language modeling (MLM), BERT variants, or replacement token detection (RTD) ELECTRA perform better. AraGPT2 is trained using a causal language modeling objective, which appears to be effective for sentence completion and language production tasks but not for classification tasks. More details about the available language model for Arabic are shown in Table 1.

The effect of parameters with different input layers, hidden layers, and attention heads on the performance of downstream tasks depends on the number of parameters used in training along with layers, hidden layers, and attention heads. Several models utilized a very low number of parameters outperform models with higher parameters. With less parameters, different combinations are also used such as those with less parameters but were pre-trained with deep hidden layers. More experiments in the side of model architecture and the effect of it in the result of downstream task need to be take attention for develop the field of Arabic PLM.

### Data Sets and Corpus

Annotated data sets are required for training a model in supervised learning approaches for text classification. Furthermore, the fine-tuning task requires label data once a transformer model has been pre-trained. Only few of these can be found in the literature in the field of emotion detection from text. Furthermore, the size of the datasets described is extremely limited. Due to the fact that emotion detection in Arabic text is a relatively new research domain, there are only few data sets available for this task; for example, El Gohary et al. (2013) annotated a data set derived from children's stories. The information was split into 2,514 non-overlapping sequential sentences from 100 articles. When annotating data, six emotion classes were used: surprise, disgust, rage, fear, sadness, and happiness. Additionally, a neutral class was created for sentences that did not communicate any emotion. For testing, another 35 documents were used. Each phrase was classified using a vocabulary that mapped individual terms to the six target categories. Emotional classes and phrases were represented as vectors, with each vector consisting of emotional word co-occurrence frequencies. To determine the proper class for each sentence, the cosine similarity metric was employed to compute the similarity between sentences and classes. If a sentence's cosine similarity to all emotional class vectors did not surpass a particular threshold, it was deemed neutral.

ArSEL, a large-scale Arabic Sentiment and Emotion Lexicon, was provided in Badaro et al. (2018b) as an extension of ArSenL (Badaro et al., 2014), with the addition of eight emotion scores to most ArSenL Arabic lemmas. EmoWordNet (Badaro et al., 2018a), a WordNet-based English emotion lexicon, was used to obtain emotion ratings. The researchers produced a large-scale mood and emotion lexicon in Arabic, which contained over 32,000 terms. Their language is based on ArSenL, an existing Arabic sentiment lexicon. They used the SeEval 2018 shared task to test their lexicon and reported an encouraging accuracy.

A study by Abdul-Mageed and Ungar (2017) used a deep learning (DL) approach for emotion detection in text and created EmoNet, a very large data set for fine-grained emotions that is claimed to be the largest data set developed from tweets; details of the dataset are presented in Table 2. In the study by Al-Khatib and El-Beltagy (2017), they constructed and balanced a data set for Arabic emotions, which was compiled from a variety of sources, one of which being tweets scraped using Twitter's search API. Egypt's geolocation was used to filter the data. These tweets were gathered over the course of a month and are primarily in Egyptian. Furthermore, happiness, anger, pity, sadness, fear, surprise, love, and none were the emotion categories. To make the process of emotion annotation easier, they used a web-based front end.

**Table 2:** Emotion data set available for Arabic language.

**Ref. no**	**Emotion dataset details**		
	**Name**	**Description**	**Size**
Badaro et al. (2018b)	ArSEL	ArSEL is an Arabic sentiment and emotion lexicon designed to supplement the publicly available Arabic Sentiment Lexicon, ArSenL, and provide a large-scale lexicon with emotion and sentiment labels for practically every lemma in ArSenL.	32,196 Arabic lemmas were annotated with sentiment and emotion scores at the same time.
Al-Khatib and El-Beltagy (2017)	AETD	Egyptian dialect tweets constitute the dataset. Anger, fear, happiness, love, sadness, surprise, sympathy, or none were used to categorize the tweets.	The total number of tweets is 10,065
Almahdawi and Teahan (2019)	IAEDS	This corpus is consisting of Iraqi dialect Facebook postings. It is divided into six datasets, each of which contains instances of Ekman's basic emotions.	1,365 posts from Facebook
Mohammad et al. (2018)	SemEval-2018	This opinion corpus consists of tweets labeled as neutral or as one or more of 11 emotions include anger, anticipation, disgust, fear, happiness, love, optimism, pessimism, sadness, surprise, and trust.	4,381 tweets
Saad (2015)	Emotion-lexicon	Arabic and English emotion lexicon, each entry in this lexicon annotated with one of Ekman's basic emotions.	Each emotion label has 3,207 Arabic words associated with it
Abdul-Mageed et al. (2016)	DINA	Tweets dataset annotated by the following emotions, happiness, sadness, anger, disgust, surprise, and fear. The tweets were gathered by utilizing a set of seeds to query Twitter for each class.	3,000 tweets
Alhuzali et al. (2018)	LAMA-DINA	Dataset for MSA and AD emotion, the tweets were labeled with the Plutchik emotions.	9,064 tweets
Yang et al. (2020)	SenWave	Collected tweets annotated for the task of fine-grained sentiment analysis with 11 emotions including optimistic, thankful, empathetic, pessimistic, anxious, sad, annoyed, denial, official report, surprise, and joking.	10 K Arabic and English tweets
Al-Laith and Alenezi (2021)	AraEmoCorpus	The corpus contains Arabic tweets tagged with emotion categories: anger, disgust, fear, joy, sadness, and surprise.	5.5 million Arabic tweets
Shakil et al. (2021)	AEELex	Tweets of users living in Mecca and Riyadh were collected, Arabic English emotion lexicon was classified on the basis of Plutchik's eight basic emotion categories.	35,383 tweets.

A multi-dialect data set for Arabic emotion analysis named DINA was created in Abdul-Mageed et al. (2016), which was one of the earliest attempts on building datasets for Arabic emotions. A seed list of Arabic emotions based on the Ekman classification of six emotions is used to collect the data set. This seed list was used to query the Twitter API. Then, 500 tweets were chosen for each of the six emotions, giving a data set of 3,000 tweets. Human annotators manually annotated the data set. The annotators were asked to determine the emotion first, then the intensity of the emotion using a scale of zero, weak, fair, or strong for each of the six emotions. In addition, Alhuzali et al. (2018) created a new data set of Arabic tweets for emotion recognition. The Plutchik primary emotions are anger, anticipation, disgust, fear, joy, sadness, surprise, and trust, and they use a list of Arabic seeds to represent each of them. After that, two data sets were created. The LAMA dataset contains 8,000 tweets, 1,000 for each of the eight emotions, which were manually annotated by four annotators. This data set had 7,268 tweets after cleaning and deleting duplicates. Using the same seed list of eight emotions, the second dataset, LAMA-DIST, was created by distant supervision. The final data set contains 182,690 tweets after cleaning and deleting duplicates and tweets with less than five words.

A study by Al-Khatib and El-Beltagy (2017) made another attempt to give an annotated data set of Arabic tweets for emotions, with a data set of 10,000 tweets annotated for eight emotions: joy, happiness, rage, sympathy, sadness, fear, surprise, love, and none. Human annotators manually annotated the data set; more details about the data set are shown in Table 2. Moreover, Hussien et al. (2016) presented an emoji-based automatic technique for emotion annotation. The feasibility of the proposed approach, which was evaluated using two classifiers, support vector machine (SVM) and multinomial naive Bayes (MNB), was demonstrated by a comparison with a manually annotated data set. The result of the experiments reveals that SVM and MNB-based automatic labeling systems outperform manual labeling techniques. A study by Almahdawi and Teahan (2019) introduced the Iraqi Arabic Emotion Data set (IAEDS), an Arabic dataset from Facebook posts written in the Iraqi dialect. Moreover, the data set was annotated according to Ekman's basic emotions. Recently, two studies had considered emotion in Twitter during the COVID-19 pandemic, SenWave (Yang et al., 2020) and AraEmoCorpus (Al-Laith and Alenezi, 2021), which are described in detail in Table 2.

### Available Resources and Tools

To develop or pre-train language models, different languages should have various quantities of resources and training data. High-, medium-, and low-resource languages are the three types. Languages with a lot of resources, such as English, Chinese, and Russian, have a lot of open-source resources and contents that can be used in training. As a result, NLP researchers have recently focused their efforts on developing resources, tools, and training data. There are some existing resources that can be utilized in the preprocessing and training of an Arabic language model. There were efforts aimed at making researchers' work easy, such as by automating routine workflows, cleaning, and preprocessing. Huggingface^3^ and AllenNLP^4^ are two examples of this. Majority of these tools are designed to work in English or to generalize a pipeline so it could be used with other languages. Arabic makes contributions as well, although not as much as other languages. Some promising examples are MADAMIRA (Pasha et al., 2014), Farasa (Abdelali et al., 2016), CAMeL NLP (Obeid et al., 2020), AraNLP (Althobaiti et al., 2014), ARBML (Alyafeai and Al-Shaibani, 2020), and AraNet (Abdul-Mageed et al., 2019).

MADAMIRA was developed by Pasha et al. (2014) for morphological analysis using algorithms. MADAMIRA's source code is accessible through email for educational purposes only, and it is also available *via* API and a web interface. The Research by Abdelali et al. (2016) developed Farasa, an Arabic NLP tool kit that could be used for segmentation and stemming, Named Entity Recognition (NER), Part Of Speech tagging (POS tagging), and Diacritization (Tashkeel), among other things. The tool kit was implemented in Java (Obeid et al., 2020) and provided a collection of open-source tools for an Arabic NLP in Python named CAMeL NLP. Currently, CAMeL provides utilities for pre-processing, morphological modeling, dialect identification, named entity recognition, and sentiment analysis.

Another study by Althobaiti et al. (2014) developed a Java-based tool kit, the AraNLP library, for processing Arabic text. Meanwhile, most preprocessing steps are supported, such as diacritic and punctuation removal, tokenization, sentence segmentation, POS labeling, root stemming, light stemming, and word segmentation. Additionally, ARBML (Alyafeai and Al-Shaibani, 2020) is a collection of tools that make Arabic NLP accessible through a variety of interfaces. These studies have aimed to address the NLP pipeline from dataset scraping to preprocessing, tokenization, training, and deployment. There are also three libraries, tnqeeb, tnkeeh, and tkseem, that developers can use to create tools that support Arabic NLP. The tools utilize Arabic's morphological nature to provide a variety of features that are unique to Arabic. Furthermore, ARBML uses Keras and TensorFlow to provide various training models with different techniques for both command lines and web interfaces. It also provides a pipeline from cleaning the dataset to model training and deployment, which is documented in detail as Colab Notebooks, and users can test different models directly in the browser. The source code, as well as the training notebooks, is available on GitHub.

In terms of tweets as a data source, we may point to AraNet (Abdul-Mageed et al., 2019), which is a collection of deep learning Arabic social media processing tools that analyze 15 datasets related to Arabic sentiment analysis, including MSA and AD, using the multilingual BERT model. Additionally, AraNet analyzed social media posts to predict age, dialect, gender, emotion, irony, and sentiment. By providing a single approach to test new models against AraNet predictions, AraNet has the potential to relieve challenges connected to comparing across diverse Arabic social media NLP tasks.

These tools help in segmentation, part of speech tagging, named entity recognition, discretization, and grammatical analysis, among other NLP tasks for Arabic. However, most of these tools may not take advantage of current developments in NLP. Unfortunately, open-source contributions are not frequently welcomed in Arabic tweet analysis and classification. Because they comply with Twitter's Terms of Service, many data sets were collection of tweet IDs and labels; if you tend to use, it you need to spend some time to retrieve and preprocess these tweets. Although some resources can be given on demand, this approach is still not developer-friendly and is time-consuming. Advances in the field of Arabic classification tasks, although it has great potential, are still limited, especially when we talk about tasks involving AD. In the next section, we will discuss some issues and challenges.

## Issues and Challenges in Emotion Analysis of Arabic Tweets

There are some challenges that researchers have faced in the field of Arabic tweet emotion analysis. Because of differences between AD and MSA, the dialectal amount of data on the Internet has expanded as a result of the rise of social media, and NLP tools that support MSA are not well-suited to analyze these data. In addition, a rich body of literature considers utilizing the MSA corpus to pre-train LM; the fine-tuning of such a model in tasks involving social media data produced unsatisfying results. Consequentially, in MRABERT experiments, the AraNETEmo data set was used to fine-tune MRABERT for emotion classification, and model F1-score was 75.83, while mBERT, AraBERT, and ARABERT, using the same dataset, F1-scores were 65.79, 65.68, and 67.73, respectively. This experiment highlighted the importance of pre-training a model with data from the origin where the fine-tuning task data will be. Another experiment used social media data, especially tweets, for pre-training a language model, QARiB, which was fine-tuned in the SemEval-2018 data set, Macro-averaged F1 for QARiB was 46.8, which was higher than that of other Bert-based models involved in that experiment. However, the results in the field of emotion analysis are not comparable with the results in sentiment analysis.

There is a limitation on the dialect's resources, Twitter users who generate Arabic tweets from the various region, over 25 dialects of Arabic are spread globally, fetching Arabic tweets can collect tweets from multiple dialects, the PLM need to be pre-train in dialects corpus as well as the fine-tuning, large and balance dataset for emotion classification of tweets need to develop and available for the researchers' community.

In general, the Arabic language suffers from orthographic ambiguity, in which the form of characters and spelling of words can vary depending on their context; on the other hand, emotions in short text like tweets can be ambiguous. Additionally, emotional words in Arabic can be understood differently according to the context that appeared. Similarly, finding implicit emotions in emotional text that lacks explicit emotional phrases is far more difficult than finding explicit emotions in direct, explicit text. Furthermore, Arabic is a morphologically rich language, which means that the same verb can have thousands of different forms.

As previously stated, tweets include characteristics that differentiate them from other types of writing and add to the task's complexity: the text is brief, informal, and contains misspellings, unique symbols such as emoticons and emojis, short forms of words, hashtags, and abbreviations. Emoticons may assist in the classification of emotions in text, but because they are not normal words and are not found in dictionaries, they are still considered anomalies in text. As a result, great care should be taken to ensure that they are not eliminated throughout the preprocessing step. In this section, we tried to discuss some of the challenges in the field and review some issues to help in developing the field of emotion analysis of Arabic tweets using language models.

## Conclusion

In this study, we tried to cover the available models, resources, datasets, tools for Arabic LM and emotion classification of tweets, and the available PLM that can be utilized for fine-tuning in the emotion detection task. According to the current state of the field, we discovered some issues and challenges. Regardless of the fact that we recognize that the NLP field is rapidly evolving and that both Arabic NLP researchers and practitioners recognize the importance of incorporating Arabic into language technologies, the AD still needs more effort to drive the field of social media classification tasks more in that direction.

## Author Contributions

GA and AA designed, conducted, and wrote the study. All authors contributed to the article and approved the final version.

## Conflict of Interest

The authors declare that the research was conducted in the absence of any commercial or financial relationships that could be construed as a potential conflict of interest.

## Publisher's Note

All claims expressed in this article are solely those of the authors and do not necessarily represent those of their affiliated organizations, or those of the publisher, the editors and the reviewers. Any product that may be evaluated in this article, or claim that may be made by its manufacturer, is not guaranteed or endorsed by the publisher.

## Footnotes

^1^ https://huggingface.co/aubmindlab/bert-base-arabertv02-twitter

^2^ https://github.com/KUIS-AI-Lab/Arabic-ALBERT/

^3^ https://huggingface.co

^4^ https://allennlp.org
